# Emodin Inhibition of Influenza A Virus Replication and Influenza Viral Pneumonia via the Nrf2, TLR4, p38/JNK and NF-kappaB Pathways

**DOI:** 10.3390/molecules22101754

**Published:** 2017-10-18

**Authors:** Jian-Ping Dai, Qian-Wen Wang, Yun Su, Li-Ming Gu, Ying Zhao, Xiao-Xua Chen, Cheng Chen, Wei-Zhong Li, Ge-Fei Wang, Kang-Sheng Li

**Affiliations:** 1 Department of Microbiology and Immunology, Shantou University Medical College, Shantou 515041, China; 16qwwang1@stu.edu.cn (Q.-W.W.); Yunsu112001@yahoo.com (Y.S.); glming2009@sina.cn (L.-M.G.); 15yzhao3@stu.edu.cn (Y.Z.); xxchen85@stu.edu.cn (X.-X.C.); cheny_118@stu.edu.cn (C.C.); gfwangstdx@sina.cn (G.-F.W.); liksedustdx@163.com (K.-S.L.); 2 Department of Veterinary Medicine, University of Maryland, College Park, and Virginia-Maryland Regional College of Veterinary Medicine, College Park, MD 20742, USA; wzlistdxedu@sina.cn

**Keywords:** influenza A virus, emodin, toll-like receptors (TLRs), MAPK, NF-κB, Nrf2

## Abstract

Lasting activations of toll-like receptors (TLRs), MAPK and NF-κB pathways can support influenza A virus (IAV) infection and promote pneumonia. In this study, we have investigated the effect and mechanism of action of emodin on IAV infection using qRT-PCR, western blotting, ELISA, Nrf2 luciferase reporter, siRNA and plaque inhibition assays. The results showed that emodin could significantly inhibit IAV (ST169, H1N1) replication, reduce IAV-induced expressions of TLR2/3/4/7, MyD88 and TRAF6, decrease IAV-induced phosphorylations of p38/JNK MAPK and nuclear translocation of NF-κB p65. Emodin also activated the Nrf2 pathway, decreased ROS levels, increased GSH levelss and GSH/GSSG ratio, and upregulated the activities of SOD, GR, CAT and GSH-Px after IAV infection. Suppression of Nrf2 via siRNA markedly blocked the inhibitory effects of emodin on IAV-induced activations of TLR4, p38/JNK, and NF-κB pathways and on IAV-induced production of IL-1β, IL-6 and expression of IAV M2 protein. Emodin also dramatically increased the survival rate of mice, reduced lung edema, pulmonary viral titer and inflammatory cytokines, and improved lung histopathological changes. In conclusion, emodin can inhibit IAV replication and influenza viral pneumonia, at least in part, by activating Nrf2 signaling and inhibiting IAV-induced activations of the TLR4, p38/JNK MAPK and NF-κB pathways.

## 1. Introduction

Influenza A virus (IAV) is a major respiratory pathogen that can easily mutate or reassort to generate a new strain and is annually responsible for high morbidity, mortality and huge economic loss all over the world. In 2009, a new reassortant swine-origin IAV (H1N1) strain had caused a pandemic spreading to 168 countries and causing 18,000 deaths worldwide within 1 year [1]. In 2013 in China, a new avian IAV (H7N9) strain had caused 440 cases of respiratory illness and 122 deaths within a 20-month period [2]. Currently, though there are two classes of anti-IAV drugs, M2 ion channel inhibitors and neuraminidase inhibitors, the use of these drugs is still limited because of their side effects and widespread drug resistance of IAV [3], so the development of novel anti-IAV drugs is still urgent.

IAV infection can activate toll-like receptor (TLR) signaling pathways. Lasting activation of TLR3 is reported to be harmful to IAV-induced acute pneumonia [4,5]. Activation of TLR4 can modulate IAV entry and tropism through MyD88 expression and p38 MAPK activation [6]. IAV infection and inactivated H5N1 avian IAV can induce severe oxidative stress and acute lung injury (ALI) through TLR4-TRIF-TRAF6-NF-κB signaling pathway [7]. Downstream cascades of TLRs, MAPK and NF-κB, are also reported to be required for IAV infection and replication [8,9,10,11,12]. 

Oxidative stress and activation of TLR pathways can mutually promote each other [13,14]. Oxidative stress also plays an important role in IAV replication and IAV-mediated pneumonia [15]. Nuclear factor erythroid 2-related factor 2 (Nrf2) signaling pathway is a classic anti-oxidative and anti-inflammatory pathway and also possesses antiviral activity [16]. Activation of Nrf2 pathway can inhibit the activation of TLR pathways [17], and also can reduce LPS-stimulated ROS-mediated cell surface transport of TLR4 and sequentially suppresses the downstream cascades, including MyD88/TRIF and pIkB/IRF3, and finally provides protection against sepsis [18].

Emodin (1,3,8-trihydroxy-6-methylanthraquinone), a natural anthraquinone compound from several traditional Chinese medicinal plants, such as *Polygonum multiflorum* Thunb., *Rheum palmatum* L., and *Polygoni cuspidati* Sieb. et Zucc., has been reported to possess antioxidant, anti-inflammatory, immunosuppressive, antiviral and anti-tumor activities [19,20]. Emodin also can inhibit the infections of coxsackievirus (CV), human respiratory syncytial virus (RSV), epstein-barr virus (EBV) and hepatitis B virus (HBV) [21,22,23,24,25]. In this study, we have examined the anti-IAV activity of emodin and explored its mechanism of action.

## 2. Results

### 2.1. Emodin Could Inhibit the Replication of IAV In Vitro

**Figure 1:**
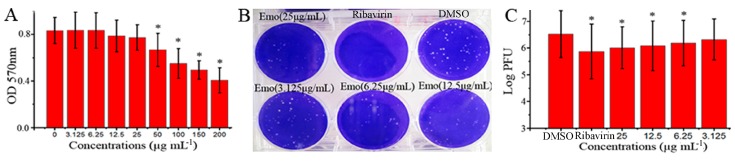
Anti-IAV activity of emodin in vitro. (**A**) The cytotoxicity of emodin on MDCK cells was determined by a MTT method. Data were mean ± SD of five independent experiments performed in triplicate. *n* = 5, * *p* < 0.05 vs. the 0 μg mL^−1^ control; (**B**,**C**) Inhibition of emodin on IAV (ST169) replication was determined by a plaque inhibition assay. In the negative control (DMSO), MDCK cells were infected with IAV (MOI = 0.001) and treated with viral growth medium (VGM) containing 0.5% (*v*/*v*) DMSO; in the positive drug control (Ribavirin) and emodin-treated (Emo) groups, MDCK cells were infected with IAV and treated with ribavirin (25 μg mL^−1^) and emodin (25, 12.5, 6.25 and 3.125 μg mL^−1^), respectively. IAV stock solution was beforehand diluted with the drug-contained VGM. After 48 h post infection (p.i.), the supernatants were collected and the titers were determined by a plaque formation assay. Data were mean ± SD of five independent experiments performed in triplicate. *n* = 5, * *p* < 0.05 vs. the DMSO control.

In this study, we first examined the anti-IAV effect of emodin *in vitro*. As shown in Figure 1A, at 25 μg mL^−1^, emodin had no cytotoxicity on MDCK cells, and the half maximal inhibitory concentration (IC_50_) of emodin was 182.95 μg mL^−1^ (Y = −0.0022X + 0.819, R^2^ = 0.9669). Additionally, at 25 μg mL^−1^, emodin also had no cytotoxicity on A549 cells (date not showed). At the concentrations ranging from 6.25 to 25 μg mL^−1^, emodin could significantly inhibit the replication of IAV (ST169, H1N1), the concentration for 50% of maximal effect (EC_50_) was 4.25 μg mL^−1^ (Y = −0.15Ln(X) + 6.581, R^2^ = 0.9833), and the antiviral index (AI, IC_50_/EC_50_) was 43.05 (Figure 1B,C). The highest concentration that showed no cytotoxicity was 25 μg mL^−1^, and we chose 25 μg mL^−1^ as the test concentration in the following pharmacological experiments.

### 2.2. Emodin Inhibited IAV-Induced Expression of TLR2/3/4/7, MyD88 and TRAF6 In Vitro

**Figure 2:**
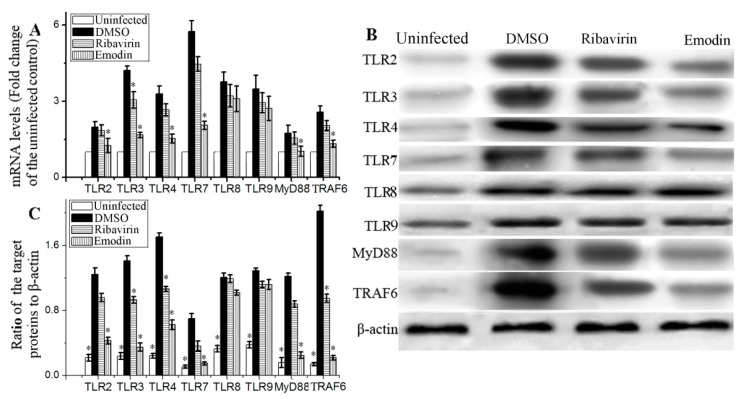
Effect of emodin on the expressions of TLRs, MyD88 and TRAF6 after IAV infection. In the uninfected group (Uninfected), A549 cells were not infected with IAV (ST169). In the negative control (DMSO), positive drug control (Ribavirin) and emodin-treated (Emodin) group, A549 cells were infected with IAV (MOI = 0.001) and treated with 0.5% (*v*/*v*) DMSO, ribavirin (25 μg mL^−1^) and emodin (25 μg mL^−1^), respectively. After 48 h p.i., the cells were harvested. (**A**) The results of qRT-PCR assay; (**B**,**C**) The results of western blotting assay. All data shown were mean ± SD of five independent experiments. *n* = 5, * *p* < 0.05 vs. the DMSO control.

TLRs-MyD88-TRAF6 pathway plays an important role in IAV-induced ALI. As showed in Figure 2, at 48 h p.i., the expressions of TLR2, TLR3, TLR4, TLR7, TLR8, TLR9, MyD88 and TRAF6 were considerably increased at both mRNA and protein levels. Emodin could significantly inhibit IAV-induced up-regulations of mRNA and protein expressions of TLR2, TLR3, TLR4, TLR7, MyD88 and TRAF6, but not for TLR8 and TLR9.

### 2.3. Emodin Inhibited IAV-Induced Activations of p38/JNK MAPKs and NF-κB Pathways In Vitro

**Figure 3:**
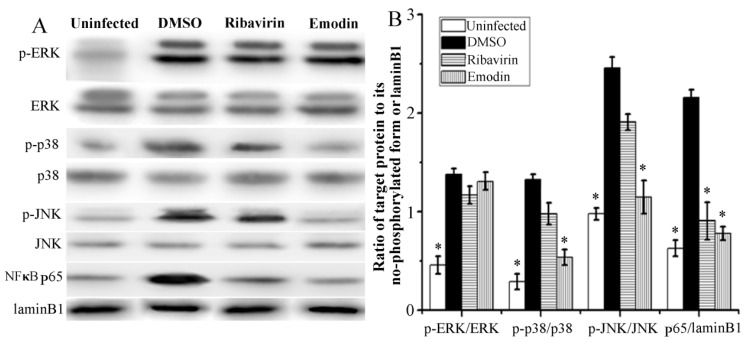
Effect of emodin on the activations of MAPKs and NF-κB signaling pathways after IAV infection. The treatments were same as those of Figure 2. Phosphorylations of ERK, p38, and JNK MAPKs and nuclear translocation of NF-κB p65 were determined by western blotting. (**A**) was the result of the western bolt, and (**B**) was the statistical result of (**A**) analyzed by Quantity One software (Bio-Rad Laboratories, Hercules, CA, USA). All data shown were mean ± SD of five independent experiments. * *p* < 0.05 vs. the DMSO control.

Downstream of TLR pathways, MAPK and NF-κB, are also involved in IAV replication and IAV-induced pneumonia. As shown in Figure 3, after IAV infection 48 h, the phosphorylations of ERK, p38, and JNK MAPKs and the nuclear translocation of NF-κB p65 were significantly increased. Emodin could significantly inhibit IAV-induced phosphorylations of p38 and JNK MAPKs and nuclear translocation of NF-κB p65, but not significant for the phosphorylation of ERK. Positive drug ribavirin also inhibited IAV-induced nuclear translocation of NF-κB p65, but not for the phosphorylations of p38, ERK and JNK MAPKs.

### 2.4. Emodin Enhanced Nrf2 Signal and Inhibited IAV-Induced Oxidative Stress

As aforementioned, IAV infection can induce severe oxidative stress, and oxidative stress can activate TLR pathways. Overactivation of TLR pathways can support IAV replication and promote IAV viral pneumonia [7,13,14,15]. Activation of Nrf2 signal can inhibit oxidative stress and suppresses the activation of TLR pathways [17]. In this study, we first detected the influence of emodin on the activation of Nrf2 signaling pathway.

**Figure 4:**
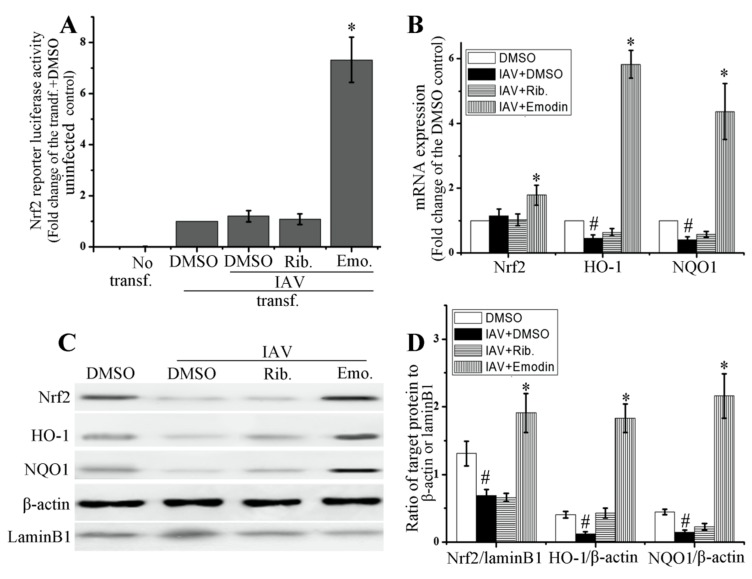
Effect of emodin on Nrf2 pathway after IAV infection. (**A**) Emodin promoted the transcription of Nrf2 luciferase reporter. A549 cells (1 × 10^6^) were transfected with ARE-luc and pRL-TK-Rluc. After 8 h, cells were infected with IAV (ST169, H1N1, MOI = 2) and treated with ribavirin (Rib. 25 μg mL^−1^) and emodin (Emo. 25 μg mL^−1^). After 24 h at 37 °C 5% CO_2_, the firefly luciferase activity was detected, normalized to that of renilla luciferase and expressed relative to the tranfected (tranf.), DMSO-treated but IAV-uninfected control; (**B**, **C** and **D**) Effect of emodin on the mRNA expressions of Nrf2, HO-1 and NQO1, nuclear translocation of Nrf2 and the protein production of HO-1 and NQO1. DMSO (0.5%, *v*/*v*), ribavirin (25 μg mL^−1^) or emodin (25 μg mL^−1^) were beforehand mixed with IAV (MOI = 0.001) and added to A549 cells for 24 h. The mRNA levels were determined by RT-qPCR. The protein levels were determined by western blotting. Data were mean ± SD of five independent experiments, *n* = 5. * *p* < 0.05 vs. the tranf. + IAV + DMSO control (**A**) or vs. the IAV + DMSO control (**B**,**D**), # *p* < 0.05 vs. the DMSO control (**B**,**D**).

As shown in Figure 4, emodin could significantly increase the transcription of Nrf2 luciferase reporter plasmid (Figure 4A), markedly enhanced mRNA expressions of Nrf2, heme oxygenase-1 (HO-1) and NAD(P)H:quinoneoxidoreductase (NQO1), two downstream effectors of Nrf2 signaling pathway (Figure 4B), and also considerably enhanced the nuclear translocation of Nrf2 and the protein productions of HO-1 and NQO1 (Figure 4C,D), after IAV infection. IAV infection could substantially decrease the production of HO-1 and NQO1.

Additionally, we also detected the effects of emodin on the production of GSH, GSSG, ROS, and on the activities of total superoxide dismutase (T-SOD), glutathione reductases (GR), catalase (CAT) and total glutathione peroxidase (GSH-Px), all of which are downstream effectors of the Nrf2 signaling pathway, after IAV infection. As shown in Table 1, IAV infection could increase the levels of ROS, decrease the level of GSH, the ratio of GSH/GSSG and the activities of T-SOD, GR, CAT and GSH-Px. Emodin could significantly improve IAV-induced oxidative stress, decreasing the levels of ROS and increasing the level of GSH, the ratio of GSH/GSSG and the activities of T-SOD, GR, CAT and GSH-Px. Ribavirin had almost no effect.

### 2.5. Nrf2 Signaling Played an Important Role in the Inhibition of Emodin on IAV-Induced Inflammation and IAV Replication

**Figure 5:**
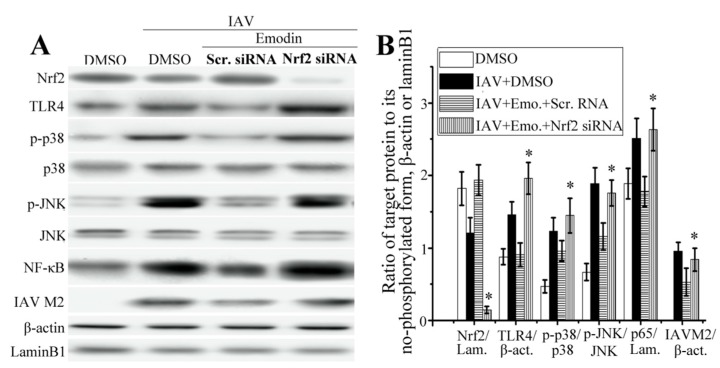

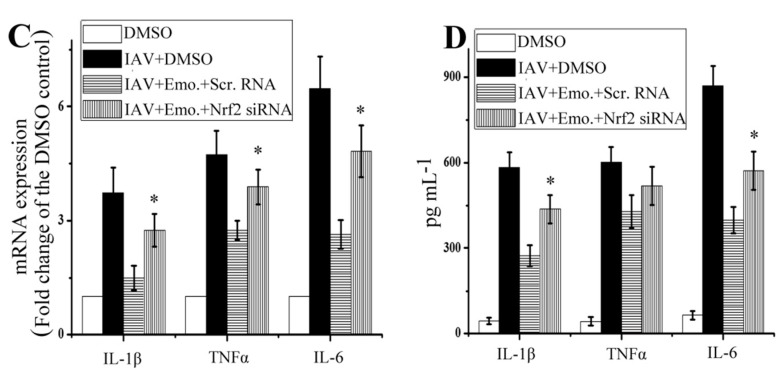
Effect of Nrf2 signaling on the inhibitory effects of emodin on IAV-induced inflammation and IAV replication. (**A**,**B**) Suppression of Nrf2 via siRNA blocked the inhibition of emodin on IAV-induced activations of TLR4, p38/JNK MAPK and NF-κB and production of IAV M2 protein; (**C**,**D**) Suppression of Nrf2 via siRNA blocked the inhibition of emodin on IAV-induced up-regulation of inflammatory cytokines. DMSO (0.5%, *v*/*v*), ribavirin (25 μg mL^−1^) or emodin (25 μg mL^−1^) were beforehand mixed with IAV (MOI = 0.001) and added to A549 cells for 24 h. Among them, two groups were pre-transfected with scramble siRNA and Nrf2-specific siRNA for 24 h, respectively. The protein levels were determined by western blotting, the production of inflammatory cytokines was determined by qRT-PCR and ELISA assays. Data were mean ± SD of five independent experiments, *n* = 5. * *p* < 0.05 vs. the scramble (Scr.) siRNA + IAV + emodin (Emo.) control.

As shown in Figure 5A,B, compared with the scramble siRNA control, suppression of Nrf2 via siRNA could markedly reduce the level of Nrf2, markedly increased the production of TLR4, phosphorylation of p38/JNK MAPK and nuclear translocation of NF-κB that were inhibited by emodin after IAV infection.

Suppression of Nrf2 via siRNA also could markedly block the inhibitory effect of emodin on the production of IAV M2 protein. Additionally, as shown in Figure 5C,D, suppression of Nrf2 via siRNA also could block the inhibition of emodin on the production of IL-1β and IL-6 after IAV infection, but not on the production of TNF-α.

### 2.6. Emodin Inhibited IAV Replication, Lung Edema and Inflammatory Response In Vivo

**Figure 6:**
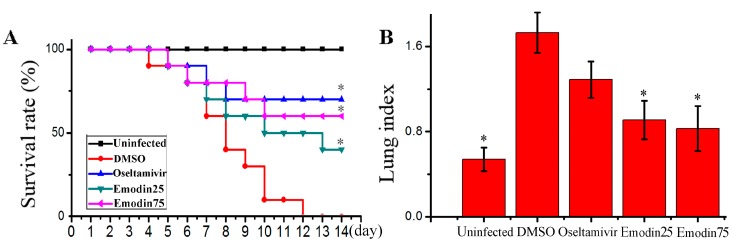

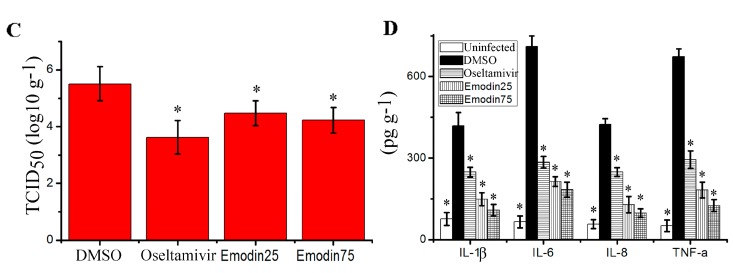
Influence of emodin on the survival rates, lung index, pulmonary viral titer and lung cytokines in mice infected with IAV (PR8). In the uninfected control (Uninfected), mice were not infected with IAV (PR8) and treated with DMSO (0.5%) by oral gavage. In the negative control (DMSO), positive drug control (Oseltamivir) and emodin-treated groups (Emodin25 and Emodin75), mice were intranasally infected with 10× MLD_50_ of IAV viruses in a 50 μL volumes, and treated with DMSO (0.5%, *v*/*v*), oseltamivir (10 mg kg^−1^ day^−1^) and emodin (25 mg kg^−1^ day^−1^ and 75 mg kg^−1^ day^−1^) by oral gavage from day −1 to day 5 p.i. (**A**) The survival rates were observed for 14 days (*n* = 10); (**B**) The lung index was evaluated by determining the percent of lung wet weight (g) to body weight (g) (lung index = lung wet weight (g) ÷ body weight (g) × 100%) at day 6 p.i. (*n* = 6); (**C**,**D**) Pulmonary viral titer and lung cytokine were determined by TCID_50_ and ELISA assays, respectively, at day 6 p.i. (*n* = 6). Data shown were mean ± SD. * *p* < 0.05 vs. the DMSO control. Chi-square (χ^2^) test was performed for A. One-way ANOVA with *post hoc* Dunnett’s test was performed for **B**, **C** and **D**.

**Figure 7:**
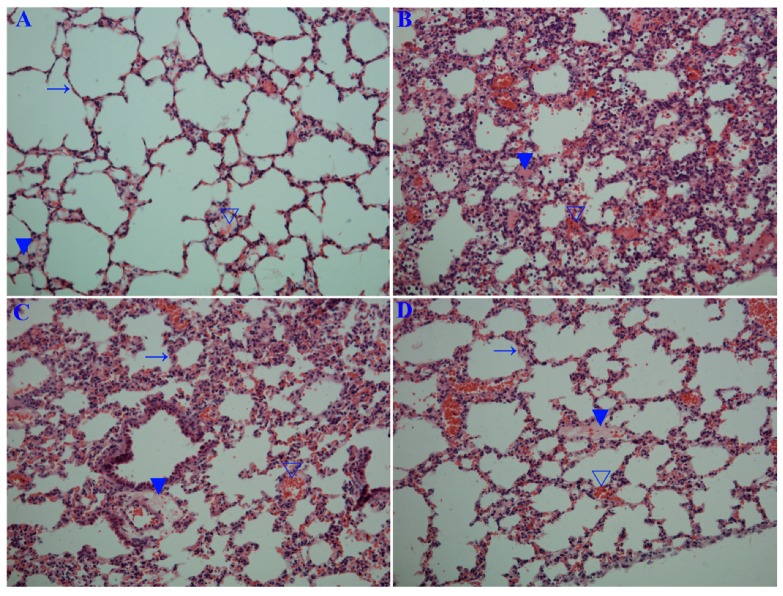

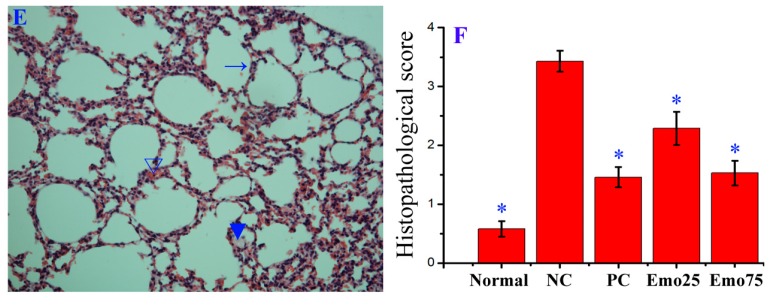
Influence of emodin on the histopathological changes. Mice were treated as Figure 6 mentioned. At day 6 p.i., six mice from each group were sacrificed. The right sides of the lungs were used in haematoxylin and eosin (H&E) staining assay. (**A**) Uninfected control (Uninfected); (**B**) Negative control (DMSO); (**C**) Positive drug control (Oseltamivir); (**D**,**E**) Emodin-treated groups (Emo25 and Emo75, respectively). (→) alveolar wall, (▼) inflammatory exudation, (∇) hemorrhage (erythrocytes). The magnification was 200×; (**F**) Histopathological score. Data shown were the mean ± SD. *n* = 6. * *p* < 0.05 vs. the DMSO control.

In this study, we determined the influence of emodin on IAV replication and its related pneumonia in mice. As shown in Figure 6, emodin could efficaciously improve the survival rate of mice infected with IAV (PR8), markedly reduced the lung index (lung edema), pulmonary viral titter, and decreased lung cytokines TNF-α, IL-1β, IL-6 and IL-8. Additionally, emodin also improved IAV-induced histopathological changes. As shown in Figure 7, after IAV infection, the lung of mice in the negative control (DMSO) showed significant alveolar exudation, destruction of the alveolar wall and alveolar hemorrhage. Positive drug oseltamivir and emodin could significantly inhibit these histopathological changes.

## 3. Discussion

Traditional Chinese Medicine (TCM) has a very long history. Now preserved TCM materials are the result of countless clinical practices during the past several thousands of years. In recent years, studies of TCM have gotten more and more attentions from the international community. Development of new antiviral agents from TCMs is believed to possess broad prospects. 

Emodin exists in many traditional medicinal Chinese herbs, such as *Polygonum multiflorum* Thunb., *Rheum palmatum* L., and *polygonum cuspidati* Sieb. et Zucc. Emodin can inhibit the infections of CV, RSV, EBV and HBV [21,22,23,24,25]. Recently, Lin et al. have reported that ethanolic extract of rhubarb (*Rheum palmatum* L.) can inhibit IAV haemagglutinin-mediated endosomal fusion [26]. Lin et al. have reported that *Polygonum cuspidati* Sieb. et Zucc. and its active components, resveratrol and emodin, can attenuate IAV infection via TLR-9-mediated IFN-β production [27]. Moreover, emodin derivative aloeemodin also can inhibit IAV infection [28]. In our study, the result showed that emodin really can significantly inhibit the replication of IAV (ST169, H1N1) (Figure 1). 

Up to now, the underlying mechanism of emodin inhibition of IAV replication and IAV-induced ALI is poorly understood. In this study, we try to explore the mechanism of action of emodin against IAV infection and IAV viral pneumonia, in an attempt to develop new anti-IAV agent. In this study, we first focused our attention on the TLR, MAPK and NF-κB signaling pathways. Nowadays it is well known that IAV infection, including H1N1, H3N2 and H5N1, can lead to the activation of TLR signaling pathways, and in turn, lasting activation of TLR signaling pathways can support IAV infection and aggravates IAV-mediated acute pneumonia [4,5,6,7]. In addition, activation of NF-κB is reported to be a prerequisite for IAV (H7N7) infection [9], and is also required for IAV (H1N1) vRNA synthesis from its cRNA [8]. Activation of p38 MAPK is also crucial for the infection and replication of IAV (H1N1), and p38 MAPK inhibitor NJK14047 can decrease IAV mRNA synthesis [29]. In a word, it is a common fact that TLRs, p38 MAPK and NF-κB signaling pathways can be activated by different IAV subtypes, including H1N1 [6,8,29], H3N2 [5], H5N1 [7] and H7N7 [9]. In our present study, we have showed that emodin can significantly inhibit IAV (H1N1)-induced expressions of TLR2, TLR3, TLR4, TLR7, MyD88 and TRAF6 (Figure 2), and can significantly inhibit IAV-induced activations of p38/JNK MAPKs and NF-κB signaling pathways (Figure 3). Based on the fact that different IAV subtypes can commonly activate TLRs, p38/JNK MAPKs and NF-κB signaling pathways, we further speculate that, besides IAV H1N1 subtype, emodin may also inhibit other IAV subtypes, such as H3N2, H5N1 and H7N7.

In fact, there are many reports about the ability of emodin to inhibit the activation of TLRs, p38/JNK MAPK and NF-κB pathways. Li A. et al. (2013) have reported that the pre- or post-treatments with emodin can significantly ameliorate LPS-induced leukocyte emigration, ROS production and the expressions of TLR4, NF-κB p65, ICAM-1, MPO and AP-1 in rat [30]. Lu Y. et al. (2013) have reported that emodin can attenuate the nuclear translocation of NF-κB p65 and attenuates the phosphorylations of ERK1/2, p38 and JNK MAPKs stimulated by phorbol 12-myristate 13-acetate (PMA) [31]. Li et al. have reported that emodin can inhibit LPS-induced activation of NF-κB, p38, ERK and JNK MAPKs signaling pathways in mice [32]. Based on these previous reports, we also speculate that the inhibitory effect of emodin on the activation of TLRs, p38/JNK MAPK and NF-κB pathways may be a common phenomenon because emodin can inhibit the activation of these pathways stimulated by LPS [30,32] or PMA [31], not only by IAV infection. 

Activation of TLR signal pathways and oxidative stress can promote each other. Ionizing radiation-induced ROS can increase the expressions of TLR2 and TLR4 through a de novo protein synthesis pathway [13]. In turn, activations of TLR2, TLR3 and TLR4 can enhance the oxidative status of intestinal epithelial cells [14]. Activation of NF-κB can drive p22^phox^ transcription, increases NADPH oxidase activity and results in oxidative stress [33]. Oxidative stress plays an important role in the pathogenesis of IAV-induced ALI/ARDS [15]. Therefore, we further explore the anti-oxidative mechanism of emodin after IAV infection.

Nrf2 signaling pathway is a classic anti-oxidative and anti-inflammatory pathway [16]. Activation of Nrf2 signaling pathway can inhibit the activation of TLR pathways [17,18]. Nrf2 can negatively regulate TLR4 innate responses through Akt-Foxo1 signal in mice [34]. HO-1 is an important downstream effector of Nrf2 signaling pathway. HO-1 activator CoPP can reduce the expressions of TLR2, TLR4, IRAK-4 and TRAF6, and markedly increases the expressions of TLR negative regulators, such as SOCS-1, IRAK-M and SHIP-1, in ischemia/reperfusion liver injury in rat [17]. Carbon monoxide (CO), a byproduct of heme catabolism by HO-1, can inhibit TLR2, TLR4, TLR5 and TLR9 signaling pathways by inhibiting ROS-induced trafficking of TLRs to lipid rafts [35]. Moreover, upregulation of Nrf2 expression can decrease IAV entry and replication in nasal epithelial cells [36], and protects human alveolar type II epithelial cells and alveolar macrophages from IAV-mediated injury by increasing the expressions of antioxidases [37].

In fact, it has been reported that emodin can remarkably enhance the expressions of Nrf-2 and HO-1, increases the activities of SOD, CAT and GSH-Px in cigarette smoke (CS)-exposed mice [38]. Emodin can activate LKB1-CaMKII-AMPK pathway, sequentially activates Nrf2 signal and enhances the productions of HO-1 and NQO1, further decreases LPS-induced activations of STATs, JNK, p38 MAPK and NF-κB, and finally reduces the productions of NO, PGE2, TNF-α and IL-6 in microglia; moreover, the anti-inflammatory effects of emodin can be reversed by transfecting with Nrf-2 and HO-1 siRNA [39]. In our study, the results show that emodin can significantly activate Nrf2 signal pathway and significantly improves IAV-induced oxidative stress (Figure 4 and Table 1). Suppression of Nrf2 via siRNA can markedly block the inhibition of emodin on IAV-induced activations of TLR4, p38/JNK MAPK and NF-κB, markedly blocks the inhibition of emodin on the production of IAV M2 protein (Figure 5). Suppression of Nrf2 via siRNA also can block the inhibition of emodin on the production of inflammatory cytokines after IAV infection. 

Finally, we have detected the effect of emodin on IAV replication and IAV-mediated inflammation in mice. The results show that emodin can increase the survival rate, reduces lung edema, viral titter and inflammatory cytokines, and improves IAV-induced histopathological changes (Figure 6 and Figure 7). Similarly to the results of our this experiment, Yin J.T. et al. (2016) have reported that emodin can alleviate lung wet-to-dry weight ratio, lung pathologic changes and lung injury in rats with sepsis in cecal ligation and puncture model [40].

**Figure 8:**
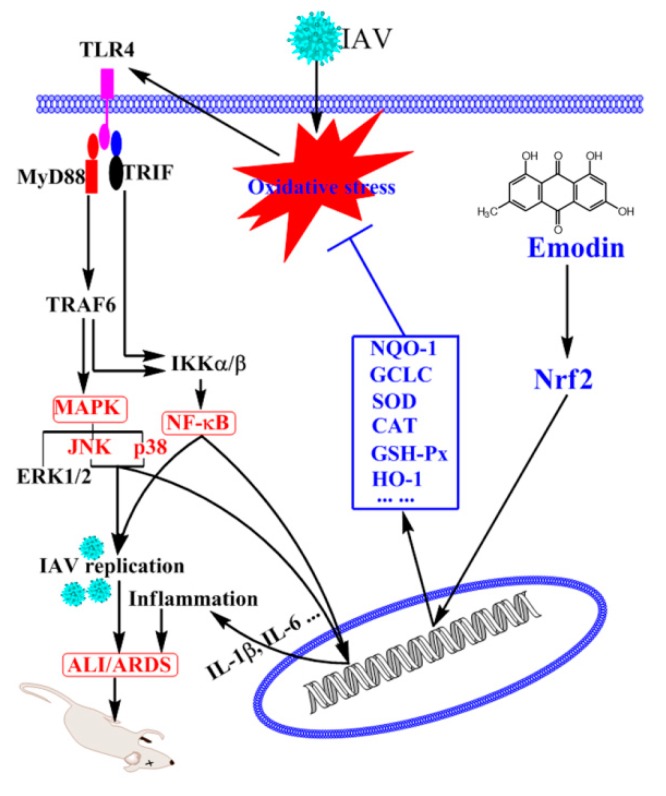
Potential mechanism of emodin to inhibit IAV replication and IAV viral pneumonia. Emodin activates Nrf2 signaling pathway, enhances the production of NQO1, GCLC, SOD, CAT, GSH-Px and HO-1 and suppresses IAV-mediated oxidative stress, which further inhibits IAV-induced activations of TLR4, p38/JNK MAPK and NF-κB pathways, leading to suppression of IAV replication and inflammatory cytokine productions and finally improving IAV-induced ALI/ARDS.

In conclusion, emodin can significantly inhibit IAV replication and IAV-mediated inflammation, and the mechanism of action may be related to its ability to activate Nrf2 signal pathway and to inhibit IAV-induced oxidative stress, activations of TLR4, p38/JNK MAPK and NF-κB signal pathways (Figure 8). 

## 4. Materials and Methods

### 4.1. Materials

Emodin (C_15_H_10_O_5_, 270.23, purity > 98%, 110756) was purchased from the Chinese Materials Research Center, National Institute for the Control of Pharmaceutical and Biological Products (Beijing, China, http://www.gjbzwz.com/zjsbzp/Emodin.html). Tosylsulfonyl phenylalanyl chloromethyl ketone (TPCK)-trypsin (4370285-1KT), ribavirin (R9644-10MG) and sulforhodamine B (SRB, 230162-5G) were purchased from Sigma-Aldrich, Inc. (St. Louis, MO, USA). Antibodies MyD88 (4283), TRAF6 (sc-8409), ERK1/2 (8867), p-ERK1/2 (13148), p-JNK (3708), JNK (4671), p-p38 (4092), p38 (14451), p65 (4764), Hrf2 (12721), HO-1 (70081) and β-actin (12262) were bought from Cell Signaling Technology^®^ Inc. (Danvers, MA, USA). Antibodies TLR2 (sc-21760), TLR3 (sc-517367), TLR4 (sc-293072), TLR7 (H-114, sc-30004), TLR8 (sc-373760), TLR9 (sc-47723), NQO1 (sc-32793), and secondary horseradish peroxidase-conjugated anti-rabbit, anti-mouse or anti-goat IgG antibody were purchased from Santa Cruz Biotechnology (Santa Cruz, CA, USA).Anti-influenza A virus M2 protein antibody (ab5416) were purchased from Abcam Inc. (Cambridge, UK). Luciferase Reporter Assay Kit was purchased from BD Biosciences Clontech (Franklin Lakes, NJ, USA). All other chemicals and solvents were commercially available and of analytical grade. 

### 4.2. Cells, Viruses and Cytotoxicity Assay

Madin-Darby canine kidney (MDCK) cells and A549 lung cancer cells were cultured in Dulbecco’s modified Eagle medium (DMEM, Invitrogen, Carlsbad, CA, USA) containing 10% fetal bovine serum (Invitrogen) and incubated in a 5% CO_2_ humidified incubator. Virus stocks of IAV subtypes A/ShanTou/169/06 (ST169, H1N1) and A/PuertoRico/8/34 (PR8, H1N1) were prepared in MDCK cells. The virus titer was determined by a plaque formation assay [41]. The cytotoxicity of emodin on the MDCK and A549 cells was determined using a MTT assay [42]. The concentration of emodin required to lower cell viability by 50% (IC_50_) was calculated using Origin 8.0 software. All experiments with IAV were performed in biosafety level 3 containment.

### 4.3. Plaque Formation and Plaque Inhibition Assays 

The viral titers of various supernatants were determined by plaque formation assay using MDCK cells as previously reported [43,44], the number of plaques (Ф > 1 mm) was counted. Plaque inhibition assay of test compounds was also performed as previously reported [45]. Briefly, the drugs were first dissolved in DMSO and then diluted with virus growth medium (VGM, MEM medium containing 1 μg mL^−1^ TPCK-trypsin and 3.2% bovine serum albumin). The final concentration of DMSO is below 0.5% (*v*/*v*). In drug-treated groups, IAV stock solution (ST169, H1N1) was diluted with this drug-contained VGM. A549 cells (1 × 10^6^) were seeded in six-well plates, after 24 h, the cells were infected with IAV (MOI = 0.001) diluted in the drug-contained VGM. After adsorption for 1 h, the cells were washed with PBS three times, and then drug-contained VGM was added. After 48 h, the supernatant was collected, and after properly diluted (ranged from 10^−2^ to 10^−5^), the viral titer was determined by plaque formation assay. 

### 4.4. TCID_50_ and Antiviral Assay by the Sulforhodamine B (SRB) Method Using CPE Reduction

IAV stock solution (ST169, H1N1) was diluted with VGM in serial dilutions, after incubation with MDCK cells for 48 h, the 50% tissue culture infective dose (TCID_50_) was calculated following the method of Reed and Muench [41,42]. Antiviral activities were further evaluated by the sulforhodamine B (SRB) method using CPE reduction [46]. Briefly, A549 cells were seeded in 96-well plates. 0.09 mL of virus suspension (50 × TCID_50_) and 0.01 mL medium containing various concentrations of the test compound were added. At 48 h, after washing, 100 μL −20 °C 70% acetone was added. After removing acetone, the plates were dried, and added 100 μL 0.4% (*w*/*v*) SRB, after washing, the plates were dried and added 100 μL 10 mM Tris-base solution. OD value was read at 562nm. Three wells were used each for the negative (virus-infected non-drug-treated) and the mock controls (non-infected non-drug-treated). 0.5% DMSO was used in each group. Percent protection of test compounds (cell viability) was calculated using the following expression:

(1) $$\mathrm{Protection}\;\mathrm{of}\;\mathrm{test}\;\mathrm{compound}(\text{\%})=\frac{\bar{OD_{test}}-\bar{OD_{Negative}}}{\bar{OD_{Mock}}-\bar{OD_{Negative}}}\times 100\%$$

The concentration providing 50% protection was defined as the EC_50_. Antiviral index (AI) was defined as IC_50_/EC_50_. 

### 4.5. Transfection and Luciferase Assay

A549 cells were transfected with Nrf2 luciferase reporter plasmid DNA using lipofectamine 2000 reagent in antibiotic free medium. After 8 h incubation at 37 °C, cells were washed with phosphate buffered saline (PBS) and virus (ST169, H1NH) was introduced (MOI = 2.0). After infection, cells were grown in VGM medium. At the same time, cells were treated with test drugs and incubated for 24 h. Transfection efficiency was normalized by co-transfection of Renilla luciferase reporter plasmid pRL-TK Vector. Luciferase activity was determined following the instruments of luciferase Reporter Assay Kit (BD Biosciences Clontech). Activity of luciferase was normalized to that of renilla luciferase and expressed as fold change to the tranfected (tranf.)-DMSO-treated but IAV-uninfected control. DMSO (<0.5% (*v*/*v*)) was used in each group to dissolve drugs. 

### 4.6. Quantitative Real Time RT-PCR (qRT-PCR)

The total RNA was extracted using Trizol^®^ Plus RNA purification kit (Invitrogen). DNA contamination in the total RNA was removed with the addition of DNase I (Invitrogen). Total RNA was eluted in nuclease-free water and quantified spectrophotometrically at 260 and 280 nm. First-strand cDNA was synthesized from 1000 ng total RNA with oligodT primer and SuperScript III reverse transcriptase kit (Invitrogen). Real-time PCR reaction mixture contained 2.5 μL of cDNA, 200 nM of each primer in SYBR Green I master mix (Invitrogen). Primers are listed in Supplementary Materials Table S1. The results were expressed as 2^−ΔΔCt^ [43].

### 4.7. Western Blotting

Western blotting was performed as previously reported [43,45,47]. Protein extracts were prepared by using RIPA lysis buffer (ShangHai Biocolor BioScience Technology Company, Shanghai, China) with a phosphatase inhibitor and a protease inhibitor. To detect the nuclear translocation of NF-κB p65 and Nrf2, nucleic protein was extracted using a EpiQuik Nuclear Extraction Kit (^#^OP-0002-1, Epigentek, Wuhan, China). Protein concentration was determined by BCA assay (Thermo Scientific, Rockford, IL, USA). Protein extracts was separated by SDS-PAGE and electrophoretically transferred onto polyvinylidene fluoride membranes (Millipore, Bedford, NY, USA). After blocking with 5% non-fat dry milk in Tris-buffered saline, membranes were incubated overnight with primary antibodies. Subsequently, a secondary horseradish peroxidase- conjugated anti-rabbit, anti-mouse or anti-goat IgG antibody was applied, and then specific bands were visualized using the ECL detection kit (^#^32132, Thermo Fisher Scientific™, Cleveland, OH, USA). β-actin was used as an invariant control for total proteins, and lamin B1 was used as a control for nuclear proteins. Protein bands intensities were analyzed by Quantity One software (Bio-Rad Laboratories, Hercules, CA, USA). 

### 4.8. ELISA Assay

Cell culture supernatants and lung tissues were collected and frozen at −80 °C. Cytokines were quantified by specific ELISA kits following the manufacturer’s instructions. Human IL-1β (DKW12-3012/-2012), human IL-6 (DKW12-1060/-2060) and human TNF-α (DKW12-1720/-2720) ELISA Kits were purchased from Dakewe Biological Technology Co., Ltd. (Beijing, China). Mouse IL-1β ELISA Kit (PI301), mouse IL-6 ELISA Kit (PI326) and mouse IL-TNFα ELISA Kit (PT512) were purchased from Beyotime Institute of Biotechnology (Shanghai, China). Mouse IL-8 ELISA Kit (MBS261967) were purchased from MyBioSource Bio Co., Ltd. (Vancouver, BC, Canada).

### 4.9. Antioxidant Assay

GSH and GSSG Assay Kit (^#^S0053) using TDNB method, Reactive Oxygen Species (ROS) Assay Kit (^#^S0033) using 2′,7′-dichlorofluorescein diacetate (DCFH-DA) method, Total Superoxide Dismutase (SOD) Assay Kit using WST-8 method (^#^S0101), Glutathione Reductases (GR) Assay Kit using TDNB method(^#^S0055), Catalase (CAT) Assay Kit (^#^S0051), Total Glutathione Peroxidase (GSH-PX) Assay Kit (^#^S0058) were purchased from Beyotime Institute of Biotechnology and performed as previously reported [41]. Briefly, A549 cells were infected with IAV (ST169, MOI = 0.001) and treated with ribavirin (25 μg mL^−1^) and emodin (25 μg mL^−1^). After 48 h, the cells were collected and used in the antioxidant assays following the manufacturer’s protocol. The protein level of each sample was also measured. 

### 4.10. siRNA Assay

Specific human Nrf2 siRNA (sc-37030) and control scramble siRNA (sc-36869) were purchased from Santa Cruz Biotechnology. A549 cells (1 × 10^6^) were transfected with Nrf2-specific siRNA or control siRNA using the X-treme GENE siRNA Transfection Reagent (Roche, Basel, Switzerland) according to the manufacturer’s instructions. After 24 h, the cells were infected with IAV (ST169, H1N1, MOI = 0.001) and treated with ribavirin and emodin at 37 °C 5% CO_2_. After 24 h, the protein levels were determined by western blotting, the production of inflammatory cytokines was determined by qRT-PCR and ELISA assays. 

### 4.11. In Vivo Study

All animal experiments were performed in accordance with the ARRIVE guidelines [48]. All experimental procedures were approved by the Institutional Animal Care and Use Committee (IACUC) of Shantou University (authorization number: SUMC2017-083). All efforts were made to minimize suffering. This model of IAV infection in mice has been put into use for several years [49]. One hundred male and female C57BL/6J mice (20 ± 2 g; 6–8 weeks; specified pathogen free (SPF)) were purchased from Shanghai Slack Laboratory Animal Co. Ltd. (Shanghai, China). Animals were housed in a specific pathogen-free facility containing standard bedding (8 mice per case) with 12-h light-dark cycles (7 to 19 h, temperature (22 ± 2 °C), humidity (40–70%), controlled ventilation) and fed with standard irradiated pellet food and sterile water *ad libitum*. Before experiments, mice were fed for 7 days for acclimation. 

In the preliminary test, twenty mice were used to determine the 50% mouse lethal dose (MLD_50_) by the method of Reed and Muench and the test doses of emodin, which we had also referred to the previous reports [40,50,51]. During experiment, 80 mice were randomly divided into 5 groups using the random number table and anesthetized by intraperitoneal injection of ketamine (100 mg/kg).

- In the uninfected control (Uninfected, *n* = 16), mice were not infected with IAV (PR8) virus but shamed with VGM medium in a 50 μl volumes intranasally, and treated with DMSO (0.5%) by oral gavage.
- In negative control (DMSO, *n* = 16), mice were intranasally infected with 10× MLD_50_ of IAV (PR8) viruses in a 50 μL volumes, and treated with DMSO (0.5%) by oral gavage.
- In positive drug control (Oseltamivir, *n* = 16), mice were intranasally infected with 10× MLD_50_ of IAV (PR8) viruses in a 50 μL volumes, and treated with oseltamivir (10 mg kg^−1^ day^−1^) by oral gavage.
- In emodin-treated groups (Emodin25 and Emodin75, *n* = 16 in each group), mice were intranasally infected with 10× MLD_50_ of IAV (PR8) viruses in a 50 μL volumes, and treated with emodin (25 mg kg^−1^ day^−1^ and 75 mg kg^−1^ day^−1^, respectively) by oral gavage.

DMSO (0.5%), oseltamivir and emodin were given twice a day (at 12-h intervals) for 6 days, starting 24 h after randomly grouping and before virus exposure. The body weights, symptoms and survivals of each group (*n* = 10) were monitored daily for 14 days after virus inoculation. For humane endpoint, animals were immediately euthanized when their weights reduced 30% and displayed with obvious ruffled fur and reduced mobility. At day 6 p.i., another six mice in each group (*n* = 6) were euthanized by cervical dislocation. The collected wet lungs were weighted and lung index was assessed by determining the percent of lung wet weight (g) to body weight (g) (lung index = lung wet weight (g) ÷ body weight (g) × 100%).Then collected lungs were separated into two sets, the right lungs were fixed in 10% formalin, and the left lungs were frozen at −80 °C. To determine the viral load and target proteins in lung, the left lungs were homogenized in 1 mL of cold MEM medium, and the protein levels were measured using the BioRad protein assay kit. Viral titer in each group was determined by TCID_50_ assay. Cytokines in homogenized lung specimens were determined by ELISA assay. The unit was corrected according to the amount of total protein in each sample. The duration of the experiment was 15 days after IAV infection. At the end of experiment, all mice were euthanized by cervical dislocation.

To examine pathological changes, the right side of the lung was embedded in paraffin, sectioned at 4 μm for haematoxylin and eosin (H&E) staining. The severity of histological changes scored under light microscopy according to a semiquantitative scoring system: 0 (no damage); 1 (diffuse reaction in alveolar walls, primarily neutrophilic, no thickening of alveolar walls, congested alveolar space in <1/4 of the field, no hemorrhage); 2 (diffuse presence of neutrophilic and mononuclear in alveolar walls with slight thickening, congested alveolar space in <1/4–1/3 of the field, at least five erythrocytes per alveolus in one to five alveoli); 3 (distinct two or three times thickening of alveolar walls due to presence of inflammatory cells, congested alveolar space in 1/3–2/3 of the field, at least five erythrocytes in five to ten alveoli); 4 (alveolar wall thickening with up to 50% of lung consolidated, congested alveolar space in >2/3 of the field, at least five erythrocytes in >10 alveoli). Each slide was assessed by two separate investigators in a blinded manner. To generate the lung injury score, a total of 20 fields were randomly observed at 200× magnification on each slide, and 10 slides were randomly detected for each sample. Representative images are shown. Additionally, to detect infiltrating neutrophilic and mononuclear, 400× magnification was used [52]. 

### 4.12. Statistical Analysis

The data and statistical analyses in this study complied with the recommendations on experimental design and analyses in pharmacology [53]. The statistical significance of comparisons between treated groups was assessed by Student’s *t*-test, one-way ANOVA with post hoc Dunnett’s test, Chi-square (χ^2^) test or Kruskal-Wallis H test using SPSS16.0 software. Data were presented as mean ± SD. Results were considered statistically different when the *p* values were equal to or less than 0.05.

## Figures and Tables

**Table 1:** Inhibition of emodin on IAV-induced oxidant stress.

**Groups**	**GSH (nmol mg Protein^−1^)**	**GSSG (nmol mg Protein^−1^)**	**GSH/GSSG (Ratio)**	**ROS (Fold Change of Uninfected Group)**
Uninfected	45.42 ± 5.33 *	3.07 ± 0.93 *	14.79 ± 0.93 *	1.00 ± 0.00
DMSO	20.48 ± 4.63	4.22 ± 0.81	4.85 ± 0.51	1.92 ± 0.17
Ribavirin	25.78 ± 5.46	3.78 ± 0.72	6.82 ± 0.62 *	1.81 ± 0.11
Emodin	33.32 ± 4.59 *	3.87 ± 0.83	8.61 ± 0.74 *	1.46 ± 0.15 *
**Groups**	**T-SOD (U mg Protein^−1^)**	**GR (U mg Protein^−1^)**	**CAT (U mg Protein^−1^)**	**GSH-Px (mU mg Protein^−1^)**
Uninfected	11.65 ± 2.63 *	11.41 ± 1.24 *	41.38 ± 5.52 *	5.21 ± 0.84 *
DMSO	4.93 ± 0.82	2.03 ± 0.52	19.43± 2.63	1.93 ± 0.27
Ribavirin	4.91 ± 0.73	2.47 ± 0.59	27.92 ± 3.16 *	2.28 ± 0.42
Emodin	7.82 ± 0.92 *	5.27 ± 0.63 *	33.64 ± 3.27 *	3.94 ± 0.49 *

In the uninfected group (Uninfected), A549 cells were not infected with IAV (ST169). In the negative control (DMSO), positive drug control (Ribavirin) and emodin-treated (Emodin) group, A549 cells were infected with IAV (MOI = 0.001) and treated with 0.5% (*v*/*v*) DMSO, ribavirin (25 μg mL^−1^) and emodin (25 μg mL^−1^), respectively. MOI = 0.001, incubation time was 48 h. Data were mean ± SD of five independent experiments performed in triplicate, *n* = 5. * *p* < 0.05 vs. the DMSO control.

## Supplementary Materials

Supplementary materials are available online.
