# Supplementary material for: Emodin Inhibition of Influenza A Virus Replication and Influenza Viral Pneumonia via the Nrf2, TLR4, p38/JNK and NF-kappaB Pathways
Source: Molecules. 2017 Oct 18;22(10):1754. doi: 10.3390/molecules22101754 (PMC6151665; doi:10.3390/molecules22101754)
Supplement: Supplementary file 1 [file molecules-22-01754-s001.pdf]

Supplementary Materials Table S1. The sequences of primers used in qRT-PCR assay.

genes	Forward (5'→3')	Reverse (5'→3')
hTLR2	ATCCTCCAATCAGGCTTCTCT	GGACAGGTCAAGGCTTTTTACA
hTLR3	TTGCCTTGTATCTACTTTTGGG	TCAACACTGTTATGTTTGTGGGT
hTLR4	TGGATACGTTTCCTTATAAG	GAAATGGAGGCACCCCTT
hTLR7	TCCTTGGGGCTAGATGGTTTC	TCCACGATCACATGGTTCTTTG
hTLR8	ATGTTCCTTCAGTCGTCAATGC	TTGCTGCACTCTGCAATAACT
hTLR9	CTGCCTTCCTACCCTGTGAG	GGATGCGGTTGGAGGACAA
hMyD88	GCACATGGGCACATACAGAC	GACATGGTTAGGCTCCCTCA
hTRAF6	AGGGACCCAGCTTTCTTTGT	GCCAAGTGATTCCTCTGCAT
hNrf2	GGCGTTAGAAAGCATCCTTCC	GCAGAGGGCACACTCAAAGT
hHO-1	AAGACTGCGTTCCTGCTCAAC	AAAGCCCTACAGCAACTGTCG
hNQO1	GAAGAGCACTGATCGTACTGGC	GGATACTGAAAGTTCGCAGGG
hIL-1β	GCAATGAGGATGACTTGTTCTTTG	CAGAGGTCCAGGTCCTGGAA
hIL-6	AGCCACTCACCTCTTCAGAAC	ACATGTCTCCTTTCTCAGGGC
hTNF-α	CCTCTCTCTAATCAGCCCTCTG	GAGGACCTGGGAGTAGATGAG
hβ-actin	AGTTGCGTTACACCCTTTCTTG	CACCTTCACCGTTCCAGTTTT
IAV M1	GGACTGCAGCGTAGACGCTT	CATCCTGTTGTATATGAGGCCCAT
